# Two New *Aspergillus flavus* Reference Genomes Reveal a Large Insertion Potentially Contributing to Isolate Stress Tolerance and Aflatoxin Production

**DOI:** 10.1534/g3.120.401405

**Published:** 2020-08-18

**Authors:** Jake C. Fountain, Josh P. Clevenger, Brian Nadon, Ramey C. Youngblood, Walid Korani, Perng-Kuang Chang, Dakota Starr, Hui Wang, Benjamin Isett, H. Richard Johnston, Raegan Wiggins, Gaurav Agarwal, Ye Chu, Robert C. Kemerait, Manish K. Pandey, Deepak Bhatnagar, Peggy Ozias-Akins, Rajeev K. Varshney, Brian E. Scheffler, Justin N. Vaughn, Baozhu Guo

**Affiliations:** * Department of Plant Pathology, University of Georgia, Tifton, GA 31793; † USDA-ARS, Crop Protection and Management Research Unit, Tifton, GA 31793; ‡ Department of Biochemistry, Molecular Biology, Entomology, and Plant Pathology, Mississippi State University, Starkville, MS 39762; § Legume Research, Mars Incorporated, Athens, GA 30605; ** USDA-ARS, Genomics and Bioinformatics Research Unit, Stoneville, MS 38776; †† Institute for Genomics, Biocomputing, and Biotechnology, Mississippi State University, Starkville, MS 39762; ‡‡ Bioinformatics, STgenetics, Navasota, TX 77868; §§ USDA-ARS, Southern Regional Research Center, New Orleans, LA 70124; *** Department of Plant Biology, University of Georgia, Athens, GA 30605; ††† Department of Human Genetics, Emory University, Atlanta, GA 30322; ‡‡‡ Nemours Hospital for Children, Wilmington, DE 19803; §§§ Department of Horticulture and Institute of Plant Breeding, Genetics and Genomics, University of Georgia, Tifton, GA 31793; **** International Crop Research Institute for the Semi-Arid Tropics (ICRISAT), Hyderabad, Telangana 502324, India

**Keywords:** *Aspergillus flavus*, aflatoxin, reference genomes, phylogenomics, polyphyletic

## Abstract

Efforts in genome sequencing in the *Aspergillus* genus have led to the development of quality reference genomes for several important species including *A. nidulans*, *A. fumigatus*, and *A. oryzae*. However, less progress has been made for *A. flavus*. As part of the effort of the USDA-ARS Annual Aflatoxin Workshop Fungal Genome Project, the isolate NRRL3357 was sequenced and resulted in a scaffold-level genome released in 2005. Our goal has been biologically driven, focusing on two areas: isolate variation in aflatoxin production and drought stress exacerbating aflatoxin production by *A. flavus*. Therefore, we developed two reference pseudomolecule genome assemblies derived from chromosome arms for two isolates: AF13, a MAT1-2, highly stress tolerant, and highly aflatoxigenic isolate; and NRRL3357, a MAT1-1, less stress tolerant, and moderate aflatoxin producer in comparison to AF13. Here, we report these two reference-grade assemblies for these isolates through a combination of PacBio long-read sequencing and optical mapping, and coupled them with comparative, functional, and phylogenetic analyses. This analysis resulted in the identification of 153 and 45 unique genes in AF13 and NRRL3357, respectively. We also confirmed the presence of a unique 310 Kb insertion in AF13 containing 60 genes. Analysis of this insertion revealed the presence of a bZIP transcription factor, named *atfC*, which may contribute to isolate pathogenicity and stress tolerance. Phylogenomic analyses comparing these and other available assemblies also suggest that the species complex of *A. flavus* is polyphyletic.

Of the secondary metabolite biosynthetic clusters identified in fungi, there are few as well characterized as aflatoxin biosynthesis in *Aspergillus flavus* and related *Aspergillus* species. From the time of its discovery in the 1960s (Amaike and Keller 2011; Forgacs and Carll 1962), the process of aflatoxin production has been under constant investigation. Identification of the bulk of the biosynthetic pathway occurred throughout the 1990s in other species, specifically *A. nidulans* (Brown *et al.* 1996). In the early 2000s, the individual genes in the biosynthetic cluster were fully described in *A. parasiticus* and later in *A. flavus* (Yu *et al.* 2004a, 2004b). The characterization of the aflatoxin cluster, however, was only the beginning of a large scale effort to sequence the entire genome of this important pathogen to learn more about its biology, plant and human pathogenicity, and the functional regulation of the production of aflatoxin and other toxic secondary metabolites produced by *A. flavus* and related fungi.

In 2003, efforts in sequencing the *A. flavus* genome were initiated with the goal of producing a draft genome for the aflatoxigenic isolate NRRL3357, a MAT1-1, L-strain isolated from peanut in Georgia (Payne *et al.* 2006; 2007; Yu *et al.* 2008). This genome, developed through Sanger sequencing at 5x coverage, was released to the National Center for Biotechnology Information (NCBI) with 2,761 scaffolds with an N50 of 2.388 Mb and a total length of 36.892 Mb (Nierman *et al.* 2015). In 2010, the genome was further revised due to contaminant sequences identified in the dataset to a final total of 331 scaffolds in the present assembly (GCA_000006275.2). This isolate has since been adopted as “type” strain for *A. flavus* and has seen near ubiquitous usage by the aflatoxin research community as a standard isolate for biological investigation of aflatoxin production. This isolate along with others such as AF13, a highly aflatoxigenic, MAT1-2, L-strain fungus from cotton field soils in Arizona (Cotty 1989), have been used in laboratory and field evaluations of breeding germplasm for resistance to *A. flavus* colonization and reduced aflatoxin contamination (Fountain *et al.* 2019a; Guo *et al.* 1995).

In addition to NRRL3357, several other isolates of *A. flavus* have also been sequenced and used for draft *de novo* genome assemblies. In 2015, AF70, a MAT1-2, S-strain from cotton field soils in Arizona, was sequenced using an Illumina platform (GCA_000952835.1). This genome was described in Gilbert *et al.* (2018) and compared to NRRL3357, where significant polymorphisms were identified potentially affecting both secondary metabolism and morphological development between the two morphotypes (S v. L strains; S – Small Sclerotia < 400µm; L – Large Sclerotia > 400µm) and mating type loci (MAT; MAT1-1 v. MAT1-2). Gene content was similar between these two isolates with 13,487 predicted in NRRL3357 and 13,118 in AF70 as were the overall lengths of the two draft assemblies. Similar levels of distinction between S and L strains were also observed by Ohkura *et al.* (2018) who sequenced three S strains (AF12, AF70, and AZS) and three L strains (BS01, DV901, and MC04). With the advent of less expensive, more rapid, and more powerful sequencing technologies, there has been an increase in the number of *A. flavus* isolate draft genome assemblies in public databases. At the time of this publication (February 2020), there are 60 released isolate draft assemblies in the NCBI Genbank (Table S1). These draft assemblies have primarily been sequenced with Illumina platforms and have an average of 997 contigs ranging in total length from 35.094 Mb to 40.273 Mb in length (Table S1). Very recently, a chromosome-level assembly of NRRL3357 with 8 chromosomes and a length of 37.749Mb was released by the University of California, Berkeley (GCA_009017415.1; Table S1).

In addition to *A. flavus*, the genomes of other *Aspergillus* species have also been sequenced. *A. nidulans* FGSC A4, *A. oryzae* RIB40, and *A. fumigatus* Af293 were all sequenced and assembled in 2005 using Sanger technology (Galagan *et al.* 2005; Machida *et al.* 2005; Nierman *et al.* 2005; Payne *et al.* 2006). Later in 2007 the genome for *A. niger* CBS 513.88, also produced using Sanger sequencing, was released (Pel *et al.* 2007). These genomes are all comprised of 8 chromosomes. Interestingly, *A. fumigatus* and *A. nidulans* have shorter overall lengths, 29.385 Mb and 29.828 Mb, respectively, compared to the other species which have genome sizes >34 Mb. This has led to the hypothesis that these species represent either earlier evolutionary development of the species complex with additional genome content being acquired through partial genome duplications, introgressions, or horizontal gene transfer (HGT); or that these species represent a distinct evolutionary event separate from that of the *A. oryzae* lineage which contains *A. flavus* (Galagan *et al.* 2005).There are 71 other species of *Aspergillus* fungi with draft or complete genome assemblies in NCBI’s Genbank (February 2020). This abundance of information provides extensive opportunities for investigating the biology and evolutionary history of this genus of fungi. However, despite this surge in information and the importance of *A. flavus* as a threat to food safety and security (Amaike and Keller 2011), there remains no complete reference genome, defined as a genome with coverage of the entire chromosomes (with expected error and gaps present) coupled with accurate annotation of associated genes, for this and other diverse isolates of this fungus.

The currently available genomes have been invaluable for and have enabled genomics-assisted experiments including transcriptome sequencing and the characterization of genes involved in a number of primary and secondary metabolic pathways. Still, an understanding of *A. flavus* phenotypic diversity has been hindered by the lack of suitable and diverse references. In addition, since reference-guided sequencing analyses rely on their reference for the identification and annotation of putative genes for analyses, the limitation of having only a single reference assembly for *A. flavus* becomes an issue given the potential for having several hundred unique genes in different isolates as seen in the comparison of NRRL3357 and AF70 (Gilbert *et al.* 2018) or among isolates with distinct morphologies (Ohkura *et al.* 2018). Therefore, to address these concerns, and to investigate the structure and evolutionary history of this pathogen, here we present two novel chromosome arm reference genome assemblies for the *A. flavus* isolates AF13 and NRRL3357. These isolates were chosen based on two biologically-driven questions: (1) what are the causes of variation in *A. flavus* isolates’ aflatoxin production; and (2) why do these isolates exhibit contrasting responses to reactive oxygen species (ROS), reactive compounds associated with drought stress which exacerbate aflatoxin production by *A. flavus* (Fountain *et al.* 2019b; Yang *et al.*2018)?

These genomes were sequenced using PacBio sequencing and scaffolds were bridged using optical mapping to produce full chromosome arms. Comparative genomics resulted in the identification of structural variation between these isolates representing the recent evolutionary acquisition of novel genes in AF13 compared to NRRL3357. The utility of these novel reference genomes in gene annotation is also demonstrated through the refinement of splice-site identification and annotation of transcriptome datasets. Comparative analysis of these references also resulted in the identification of a novel bZIP transcription factor gene, annotated *atfC*, which may contribute to stress tolerance in *A. flavus* under drought stress conditions. Phylogenomics analyses also show that the *A. flavus* section *Flavi* is polyphyletic, and that AF13 represents a distinct but closely related sister clade of NRRL3357. These reference genomes represent a valuable asset for use by the *Aspergillus* research community, and will serve as a starting point for continuing research into the biology of these organisms, particularly for stress biology related to oxidative stress and aflatoxin production.

## Materials and Methods

### Isolate collection and culturing

For isolates used for genome sequencing and assembly, NRRL3357 was obtained from the USDA-ARS Northern Regional Research Center, Peoria, IL, USA; and AF13 was obtained from Kenneth Damann, Department of Plant Pathology and Crop Physiology, Louisiana State University Agricultural Center, Baton Rouge, LA, USA. Additional isolates collected for re-sequencing and comparisons are as follows. A1, A9, AF36 (NRRL18543), Afla-Guard (NRRL21882), Tox4, VCG1, and VCG4 were obtained from K. Damann. K49 (NRRL30797) and K54A were obtained from Hamed Abbas, USDA-ARS Biological Control of Pests Research Unit, Stoneville, MS, USA. All isolates were received on potato dextrose agar (PDA) plates, and were transferred to V8 agar (20% V8, 1% CaCO_3_, 3% agar) to stimulate conidiation. For long term storage, 5 – 6 agar plugs were taken from the growing edge of the plates, and placed into amber vials containing 5 mL of either sterile water or 20% glycerol and stored at 4° and -20°, respectively. These conidial suspensions (∼10^7^ conidia/mL) were used as inoculum for subsequent experiments. Phenotypic differences between AF13 and NRRL3357 were evaluated on V8 agar. Differences in conidia production between these isolates were evaluated by washing V8 agar plates of each isolate with 25mL of 0.1% (v/v) Tween 20, and the concentration obtained for each conidial suspension was measured using a hemocytometer. This evaluation was performed three times.

### Standard and high molecular weight DNA isolation

For short read sequencing of the isolate collection, a normal CTAB DNA isolation was done as follows. Each isolate was cultured in yeast extract – sucrose (YES, 2% yeast extract, 1% sucrose) for five days at 30° in the dark. Mycelial mats from each culture were collected and ground in a chilled mortar and pestle with liquid nitrogen. The ground mycelia (1-2 g) was then combined with 15 mL of CTAB extraction buffer (0.1M Tris pH 8.0, 1.4M NaCl, 20mM EDTA, 2% (w/v) CTAB, 4% (w/v) polyvinylpyrrolidone (PVP-40), and 0.5% (v/v) β-mercaptoethanol), mixed by inversion, and incubated in a water bath at 65° for 45 min with occasional inversion. The lysate was then combined with 15 mL of chloroform:isoamyl alcohol (24:1), mixed by inversion, and centrifuged at 8,000 × g for 15 min at 4°. The upper phase was then transferred to a new 50 mL centrifuge tube. The chloroform separation was then performed a second time, and the upper phase was then combined with one volume of cold isopropanol for DNA precipitation. The DNA was then pelleted by centrifuging at 8,000 × g for 15 min at 4°, and washed with 70% ethanol. The pellets were then dried and suspended in 100 µL TE buffer (10mM Tris pH 8.0, 1 mM EDTA pH 8.0). RNaseA was then added to a final concentration of 5 µg/mL and the samples were incubated at 37° for 1 hr. The obtained DNA was then stored at -20° until used.

For long read sequencing of AF13 and NRRL3357, high molecular weight (HMW) DNA was isolated using a modified version of the CTAB protocol. Ground mycelium (1-2 g) was combined with 15 mL CTAB buffer as previously described, but with the addition of 75 µL proteinase K (20 mg/mL) to each sample to improve cell lysis along with the addition of 20 µL RNaseA (10 mg/mL). The samples were then incubated at 60° for 45 min with occasional gentle agitation. The temperature was increased to 70° for 15 min to begin inactivating proteinase K. The lysate was combined with 15 mL of phenol:chloroform:isoamyl alcohol (25:24:1), mixed by gentle inversion, and centrifuged at 8,000 × g for 15 min at 4°. The upper phase was then transferred to a new 50 mL centrifuge tube using a large bore pipet, and was combined with 15 mL of chloroform:isoamyl alcohol (24:1), mixed by gentle inversion, and again centrifuged. The resultant upper aqueous phase was transferred to a new tube and DNA was precipitated with one volume of cold isopropanol and 2 mL 7.5 M ammonium acetate. The DNA was pelleted by centrifugation and washed with 70% ethanol. After drying, the pelleted DNA was then dissolved in 500 µL TE buffer and stored at -20° until use. DNA isolated using either method was quantified with both a Nanodrop ND-1000 spectrophotometer (ThermoFisher, Waltham, MA, USA) and a Qubit 3.0 fluorometer (ThermoFisher), and checked using gel electrophoresis.

### DNA sequencing

Isolated DNA for short read sequencing was frozen and shipped to the Novogene Corporation (Sacramento, CA, USA). Sequencing was carried out as described in Fountain *et al.* (2020) using a HiSeq 4000 platform (Illumina, San Diego, CA, USA). For long read sequencing, HMW DNA from AF13 and NRRL3357 were frozen and shipped to the USDA-ARS Genomics and Bioinformatics Research Unit, Stoneville, MS, USA for sequencing. Sequencing was carried out on a PacBio RSII platform (Pacific Biosciences, Menlo Park, CA, USA). These PacBio reads were then used in conjunction with optical mapping for reference assembly construction.

### Optical mapping

In order to bridge contigs in the assembled PacBio genomes for AF13 and NRRL3357 to assemble full chromosomes, and given the lack of a published genetic map for *A. flavus*, optical mapping was performed at the Emory Integrated Genomics Core at Emory University, Atlanta, GA, USA. A modified protocol was developed for HMW DNA isolation from *A. flavus* protoplasts. The protocol used for protoplast generation and preparation was based on those used by Cary *et al.* (2006), Liu and Friesen (2012), and Yang *et al.* (2016). Briefly, conidia from each isolate were grown on V8 agar for five days. Plugs were taken from the growing edge of the generated colonies and were placed into amber vials containing 5 mL of sterile water. With this, 1 mL of each inoculum (∼10^6^ conidia/mL) was added into 250 mL of potato dextrose broth (PDB) in a 1 L media bottle which was capped and sealed with parafilm. After culturing for 12 hr at 30° in the dark, mycelia were isolated by vacuum filtration through two layers of Miracloth (Millipore-Sigma, Burlington, MA, USA). The isolated mycelia were then washed three times with sterile water and transferred to a sterile 50 mL centrifuge tube. Enzymatic digestion of the fungal cell walls was then carried out by adding 40 mL of enzyme solution to mycelia from each isolate. This digestion solution was prepared by combining 4 mL 0.2 M NaPO_4_ pH 5.8, 0.8 mL 1.0 M CaCl_2_, 2.8g NaCl, 139.48 µL β-glucuronidase (24,377 U/mL; Sigma G8420), 400 mg lysing enzyme (Sigma L1412), 100 mg driselase (Sigma D9515), and 34 mL sterile water. The solution was gently stirred for 5 – 10 min to allow the materials to completely dissolve, followed by centrifugation at 2,000 × g for 10 min at 4°, and filter sterilization of the resultant supernatant. Digestion of the mycelia was carried out over 3 hr at 30° with gentle shaking (80 rpm).

The resultant digestions were then filtered through four layers of Miracloth to separate the protoplasts from undigested mycelial fragments, and stored on ice for the remainder of the procedure. The protoplasts were then pelleted by centrifugation at 300 × g for 10 min at 4°, washed with 20 mL of mycelia wash solution (MWS; 0.7M KCl, 10mM CaCl_2_), pelleted and washed in 500 µL of cell buffer from the Bionano Prep Blood and Cell Culture DNA Isolation Kit (Bionano Genomics, San Diego, CA, USA). The protoplasts were then pelleted again and resuspended in 66 µL of cell buffer to a final concentration of at least 10^9^ protoplasts/sample (>6 µg DNA content) for use in agarose plug generation. Throughout the procedure following digestion filtration, the protoplasts were quantified and evaluated for viability using a hemocytometer and a Countess automated cell counter (ThermoFisher). For cell lysis and HMW DNA isolation, the protoplasts were then cast into agarose plugs. For each plug, 66 µL of cell suspension was combined with 40µL of molten 2% low melting point agarose, mixed with a wide bore pipette, and placed into a plug mold (Cat# 1703713, Bio-Rad, Hercules, CA, USA) at 4° for plug solidification. Proteinase K digestion, RNaseA digestion, washing, and HMW DNA isolation were then performed using the Bionano DNA isolation kit according to the manufacturer’s instructions. The integrity of the isolated HWM DNA was evaluated using pulse field gel electrophoresis (PFGE). Labeling of the HWM DNA for use in sequencing was done using the Bionano Prep DLS (Direct Label and Stain) Labeling Kit (Bionano Genomics) according to the manufacturer’s instructions. Sequencing was then carried out on a Saphyr platform (Bionano Genomics).

### Genome assembly

PacBio reads were assembled using Mecat. This assembly resulted in 16 fully contiguous sequences representing all chromosome arms (broken only by centromeric sequence). Chromosome arms were paired, and chromosome numbers assigned using collinearity with the *A. oryzae* RIB40 sequence (GCA_000184455.3), which was produced previously based on optical maps. Chromosome 6 (Chr6) and Chr2 are involved in a reciprocal translocation. We assigned the portion of the PacBio contig closest to the centromere to its respective *A. oryzae* chromosome based on the most parsimonious explanation of two chromosome breaks and translocation. Fifty “N” characters were placed between chromosome arms as a stand-in for actual centromere sequence.

### Gene annotation and presence/absence variation

The evidence-based gene prediction pipeline, MAKER, was used for genome annotation. MAKER aligns expressed sequence tags (ESTs) and protein evidence to a genome, produces *ab-intio* gene predictions, and identifies repeats (Cantarel *et al.* 2008). Expressed sequence tag and protein evidence were obtained from the *Aspergillus* Genome Database (AspGD) (Arnaud *et al.* 2012). The AspGD is a central repository for gene annotation and protein information for *Aspergillus* species. Specifically, sequences from *A. flavus* NRRL 3357 and *A. oryzae* RIB40 with no introns for all open reading frames (ORFs) were used as EST evidence and protein evidence was provided by translations of all ORFs of *A. fumigatus* Af293, *A. niger* CBS 513 88, and *A. nidulans* FGSC A4. The Augustus (Stanke *et al.* 2004) trained dataset of *A. oryzae* was used for *ab-initio* gene prediction and repeat soft-masking was performed using the *Aspergillus* repeat library from RepBase (Bao *et al.* 2015). The Galaxy tool (Afgan *et al.* 2018) version 2.31.9.1 of MAKER was run on a Galaxy SlipStream server (BioTeam Inc. Middleton, MA) to perform above mentioned MAKER pipeline. Annotation of secondary metabolite gene clusters was performed using the web-based application antiSMASH (v5.0; Blin *et al.* 2019). Annotation was performed for tRNAs using tRNAscan-SE (v2.0.5; Chan and Lowe 2019), and for rRNAs using Barrnap (v0.8; https://github.com/tseemann/barrnap, last accessed August 5, 2020).

In order to connect pre-existing annotations with new annotations and examine variation in gene content, coding sequences were extracted from GFF files. The AF13 and NRRL3357 coding sequences (CDS) was combined with the AFL1 reference transcriptome derived from NCBI_Assembly GCF_000006275.2 (JCVI-afl1-v2.0). This combined set was searched against itself using *nucmer* (version 3.1) with maxmatch flag. Results were filtered based on overall alignment length across all sub-matches in the same orientation between the pairs of sequences. If the overall alignment length was >80% of the longest sequence in the pair, then the pair was retained. This length criterion was based on manual curation and designed to cluster alternative transcripts and homologs that are likely to have very similar function. A pairwise matrix of all sequences was built using this overall alignment length as a distance criterion. *mcl* (version 14) was then used to cluster sequences based on this matrix.

### Insertion/deletions inference

Indels were identified from whole genome alignments and were polarized relative to the outgroup, *A. oryzae* RIB40, using a custom program. Columns in the whole chromosome alignments that involved >50 consecutive gaps (in any sequence) were extracted along with +/− 50 bp of flanking sequence. Gaps were analyzed further if the left and right flanking regions aligned with >90% columns being identical. If AF13 and NRRL3357 shared 95% identity in the gapped region, the structural variant (SV) was not considered further. Alternatively, if there was variation between AF13 and NRRL3357 and one matched the outgroup with >95% identity, then the event was inferred to have occurred in the non-matching sequence. This approach captured the biological reality that mutations creating long (>50 bp) SVs rarely involve only insertion or deletion of DNA but a combination of both. To that end, we also characterized the degree to which mutations represents a net gain or loss of DNA. The length of the entire gapped region was divided by the length of novel sequence introduced in the gap such that values approaching 0 are, in effect, deletions and values approaching 1 are insertions (a small number of SVs with gap values between 0.49 and 0.51 were removed after manual curation indicated these “perfectly balanced” indels represent unwarranted gap openings).

### Phylogenetic analyses

Illumina short read data were obtained from the results of the “DNA Sequencing” section above. Assembled contigs for *A. flavus* isolates 206-4, 26-3, 3-2, 40-5. 54-2, 61-4, 72-5, 78-6, 79-2, CA14, CS0504, CS1137, JAU2, NRRL21882, NRRL18543, NRRL30797, and WRRL1519 were obtained from NCBI. All lines, short reads and contigs, were aligned to the AF13 reference using BWA v 0.7.1 with standard parameters. These alignments were sorted and indexed, and read depth per position was calculated and visualized via IGVtools 2.7.2. These alignments were then used to call short indels and SNPs using the BCFtools ‘mpileup’ and ‘call’ commands (version 1.9-274-g7db9558+). The samples were treated as haploid, and a multiallelic model was used, allowing for more than 2 alleles per position to be called. These variants were filtered to exclude sites that were present in fewer than 29 lines. A phylogenetic tree was created to visualize relationships from this filtered variant data using the UPGMA method in TASSEL 5.

### RNA sequencing

To facilitate annotation of the newly developed genomes and to explore the signaling responses of *A. flavus* to drought-related oxidative stress, an RNA sequencing experiment was conducted. The AF13 isolate was cultured on V8 agar for five days, and conidia were harvested as inoculum (10^7^ conidia/mL). The isolate was cultured in 50 mL of YES liquid medium in a 125 mL Erlenmeyer flask capped with sterile cotton for 48 hr at 30° with shaking at 150 rpm. After 48 hr, hydrogen peroxide (H_2_O_2_) was added to a final concentration of 30 mM in each treated culture. Control cultures received no H_2_O_2_. Mycelia were then collected at 0, 3, 6, and 9 hr after H_2_O_2_ amendment and flash frozen in liquid nitrogen and stored at -80°. Four replicate cultures were collected at 0, 3, and 6 hr for both treated and control samples, and two replicated cultures were collected at 9 hr for both. This yielded a total of 24 samples for RNA sequencing.

The collected mycelia were ground to a fine powder using a Bullet Blender 24 (Next Advance, Troy, NY, USA), and total RNA was isolated using a RNeasy Plant Mini Kit with on-column DNase digestion (Qiagen, Hilden, Germany). Sample quantity and quality were estimated using a Nanodrop ND-1000 spectrophotometer (ThermoFisher) and gel electrophoresis. Isolated total RNA was then frozen and shipped to the Novogene Corporation for quality checks, library preparation, and sequencing. RNA integrity numbers (RINs) were measured using an Agilent 2100 Bioanalyzer (Agilent Technologies, Santa Clara, CA, USA) and samples used for sequencing had RINs > 7.0. Library preparation was done using a TruSeq Library Prep Kit (Illumina) according to the manufacturer’s instructions. Prepared libraries were quantified using a Qubit 2.0 fluorometer (ThermoFisher), and sequenced on a HiSeq 4000 platform (Illumina).

### Transcriptome analysis

Differential expression analysis was done using kallisto pseudoaligner with 10 boostrap iterations (Bray *et al.* 2016). Raw counts were then analyzed using DESeq2 (Love *et al.* 2014). Two different models were tested. First, the effect of oxidative stress was tested using the full model, gene ∼ trt + time, and the reduced model, gene ∼ trt. Second, the effect of time under stress was tested using only the 0 time point and inoculated samples using the full model, gene ∼ time, and the reduced model gene ∼1. Genes were determined to be differentially expressed with the adjusted *P* < 0.05 using a Bonferroni multiple testing correction.

### Functional characterization of atfC and isolate phenotyping

#### atfC disruption mutant generation:

Annotation of the 310 Kb insertion in AF13 identified a putative bZIP transcription factor gene homologous with *atfA* and *atfB*. This transcription factor, dubbed *atfC*, was functionally evaluated for its influence on stress tolerance and aflatoxin production. Disruption of *atfC* was carried out using a double-crossover recombination approach as previously described by Chang *et al.* (2010). Briefly, the disruption vector was constructed through the introduction of the *ptrA* (pyrithiamine (PT) resistance) marker amplified from the pPTR1 vector (TaKaRa Bio, Japan), combined with 0.9 and 0.7 kb fragments of the 5′ and 3′ ends of *atfC*, respectively, including some flanking sequences using PCR to generate the pAtfCDV vector. Protoplasts of AF13, generated as previously described (Chang *et al.* 2010), were then transformed using polyethylene glycol (PEG) as described by Horng *et al.* (1990) with minor modifications, and selected on CZ regeneration medium containing 0.6 M KCl, 5 mM (NH_4_)_2_SO_4_, and 0.1 µg PT/mL for up to 10 days at 30°. The insertion and orientation of *ptrA* into *atfC* in AF13 were then evaluated using diagnostic PCR and gel electrophoresis. Empty transformation vectors lacking the disruption construct were used as controls in the experiment. In addition to disruption, an additional copy of *atfC* along with its native promoter and terminator sequences (1.0 kb up and down-stream of the coding region) was introduced into NRRL3357 and AF13 wild type (WT) isolates to examine introduction and dosage effects, respectively. In addition to PCR amplicon size, insertions and deletions of *atfC* were also confirmed using Sanger sequencing. Overall, two isolates were identified for each event and used for downstream phenotypic characterization.

#### Phenotypic characterization and H_2_O_2_-stress tolerance:

Once obtained, the isolates along with the WTs were screened for gross morphological effects of their respective mutations on different media including Czapek-Dox agar, PDA, and V8 agar. The isolates were then examined for effects on oxidative stress tolerance by culturing them on a gradient of H_2_O_2_-amended YES liquid medium ranging from 0 – 50 mM H_2_O_2_ for seven days at 30° in the dark as previously described (Fountain *et al.* 2015a) in either stationary in 125mL Erlenmeyer flasks or with shaking at 150 rpm in 50 mL conical bottom tubes. Culture medium was also sampled from each isolate and developed using thin layer chromatography as previously described (Fountain *et al.* 2015a; 2019b) to examine for effects on aflatoxin production under increasing oxidative stress.

#### Pathogenicity and aflatoxin assays on peanut kernels:

Effects on pathogenicity and aflatoxin production *in vitro* were evaluated for the WT and disrupted isolates of AF13 using a kernel screening assay as described by Guo *et al.* (1995) with modifications. Seeds of the peanut cultivar Tifrunner with intact testa and free of visible damage were surface sterilized using UV exposure for 60 min. To examine each isolate, sterilized seeds were immersed in an inoculum containing 10^5^ conidia/mL in 0.1% (v/v) Tween 20. The seeds (four seeds per well) were then transferred to sterile 6-well cell culture plates which were then placed into moist chambers and incubated for five days at 28° in the dark. The seeds were then evaluated for visible fungal growth and conidiation as an indicator of isolate pathogenicity. The seeds were then collected, ground into powder, and placed into 2mL tubes. The tubes were weighed and a 1.0 mL solution of 5% (*w/v*) NaCl and 80% (*v/v*) methanol was added to each tube. The tubes were then vortexed, kept at room temperature for 30 min, and centrifuged at 10,000 rpm for 10 min for aflatoxin extraction. For quantification, 100 µL of extraction supernatant was added to 400 µL of HPLC-grade water in 2 mL tubes and vortexed. The resultant solution was tested for aflatoxin concentration using a VICAM Series-4EX Fluorometer (Vicam, Milford, MA, USA) with Afla B columns according to the manufacturer’s instructions. Obtained data were then normalized based on seed weight and dilution, and analyzed by ANOVA with post-hoc grouping and non-parametric transformation using R (v3.5.2).

### Data availability

Analyzed data are provided in the attached supplementary files. The assemblies and associated metadata are available through NCBI Bioproject IDs PRJNA606291 for NRRL3357 and PRJNA606266 for AF13. Genome assembly accession numbers at NCBI are GCA_014117465.1 for NRRL3357 and GCA_014117485.1 for AF13. Fungal isolates are available upon request by contacting the corresponding author. Supplemental material available at figshare: https://doi.org/10.25387/g3.12816593.

## Results

### Chromosome-level assemblies for two isolates of A. flavus

Two reference genome assemblies were generated for AF13 and NRRL3357 (Figure S1). Using PacBio sequencing, for AF13, a total of 7.73 Gb of sequencing data were generated with an average read length of 12,822 bp and read N50 of 21,750 bp. For NRRL3357, 7.97 Gb of sequencing data were generated with an average and N50 read length of 10,437 and 18,750 bp, respectively. These data were sufficient for 210 and 216X coverage for AF13 and NRRL3357. For assembly, reads >15kb in length were used yielding ∼70X coverage for each isolate assembly. Overall, 19 and 69 contigs were generated for AF13 and NRRL3357, respectively, and these contigs were then further assembled into 19 and 17 scaffolds (Table 1). When further assembled, these scaffolds approached chromosome-length assemblies generating eight pseudomolecules for each isolate (Table 2). Large variants detected between assemblies and linkage between scaffolds to generate chromosome arms were validated using Bionano optical mapping. Chromosomal assignments were based on homology and alignment with the related *A. oryzae* RIB40 genome (GCA_000184455.3). RIB40 alignments were also used to confirm scaffold ordering. Lengths of the assembled chromosomes ranged from 6.783 to 3.015 Mb for AF13 and 6.387 to 3.033 Mb for NRRL3357 (Table 2). Final lengths of the assembled genomes were 37.439 Mb for AF13 and 36.996 Mb for NRRL3357, which are comparable to those obtained for other *A. flavus* assemblies in public databases (Table S1).

**Table 1:** Assembled contig and scaffold descriptor statistics for AF13 and NRRL3357

Descriptor	AF13		NRRL3357	
	Length (Mb)	n	Length (Mb)	n
**Contigs**				
**N50**	2.579	6	1.998	7
**N60**	2.145	8	1.827	9
**N80**	1.929	11	0.659	17
**N90**	1.876	13	0.357	25
**Total**	37.599	19	38.645	69
**Average/Contig**	1.979		0.560	
**Scaffolds**				
**N50**	2.388	6	2.398	6
**N60**	2.169	8	2.114	8
**N80**	1.929	12	1.927	11
**N90**	1.876	13	1.823	13
**Total**	37.439	19	36.996	17
**Average/Scaffold**	1.979		2.179	
**Largest Scaffold**	4.615		4.517	
**Gaps**	0		0	

**Table 2:** Assembled chromosomes for AF13 and NRRL3357

	AF13			NRRL3357		
Chromosome	Length (bp)	GC (%)	Predicted Genes	Length (bp)	GC (%)	Predicted Genes
**Chr1**	6,783,352	47.90	2,146	6,386,556	48.07	2,075
**Chr2**	6,263,604	48.12	2,026	6,246,150	48.09	2,031
**Chr3**	5,029,825	48.16	1,619	5,100,955	48.02	1,636
**Chr4**	4,650,921	47.85	1,489	4,658,713	48.08	1,518
**Chr5**	4,535,909	47.61	1,483	4,453,722	48.23	1,472
**Chr6**	4,021,220	47.87	1,321	3,936,580	48.24	1,290
**Chr7**	3,015,401	48.15	933	3,033,036	47.90	941
**Chr8**	3,138,692	47.63	1,037	3,179,870	47.39	1,046
**Average/Chr**	4,679,866	47.91	1,507	4,624,448	48.00	1,501
**Unmapped (bp)**	159,798	—	—	53,376	—	—
**Total**	37,438,924	—	12,054	36,995,582	—	12,009

### Indel and structural analyses reveal a novel 310kb insertion between the assemblies

**Figure 1:**
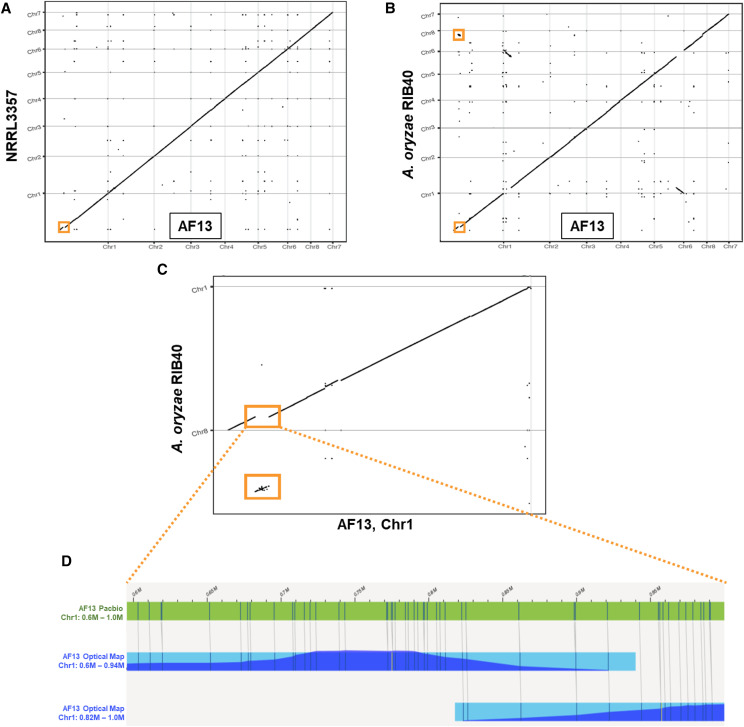
Whole genome alignment and structural confirmation using optical mapping. A. Dotplot showing a comparison between AF13 and NRRL3357. A large insertion (310 Kb) can be observed on Chromosome 1. B. Comparison between AF13 and *A. oryzae* RIB40 at the insert position (enlarged in C) clearly showed alignment to a region on *A. oryzae* Chromosome 8 for the insertion. Otherwise, the genomes shared a similar structure with the exception of a translocation on Chromosomes 6 and 2. D. Bionano optical mapping reads (blue) aligned to assembled PacBio contigs (green) show sufficient read depth in the region to confirm the presence of the insertion and validate the AF13 assembly.

Structural and indel variation between the assemblies (Table S2) was evaluated leading to the discovery of a large, 310 Kb insertion present on Chromosome 1 of AF13 ranging from 655,567 – 967,172 bp that was completely absent from NRRL3357 (Figure 1A; Figure S2). This insertion shared homology with a similarly sized region on Chromosome 8 of the *A. oryzae* RIB40 genome, but limited homology with other *Aspergilli* and Eurotiomycete fungi suggesting that this region may be either derived from *A. oryzae* by horizontal transfer, represent a degenerate version of the *A. oryzae* Chromosome 8 region, or may represent a distinct lineage of *A. flavus* following speciation from *A. oryzae* (Figure 1B,C). The presence of this insertion, however, could be the product of sequence assembly artifacts. Therefore, Bionano optical mapping was used to confirm the presence of the insertion in the AF13 genome which clearly demonstrated that the insertion was genuine (Figure 1D).

### Phylogenomics and prevalence of the 310 Kb insertion among other Aspergillus genomes

To examine the prevalence of the 310 Kb insertion within the species, available *A. flavus* genomes were collected from NCBI (Table S1) for comparative analyses with the AF13 assembly. In addition to these, the genomes of 10 additional isolates, including one *A. parasiticus* isolate, were sequenced using Illumina sequencing and used for comparative analyses (Fountain *et al.* 2020; Table S1). Based on blastn searches and structural comparisons (Figure 1) the insert was found to bear a relatively high level of similarity of a similar sized region of Chromosome 8 in the *A. oryzae* RIB40 genome. Therefore, this genome was also included in the comparative analysis.

**Figure 2:**
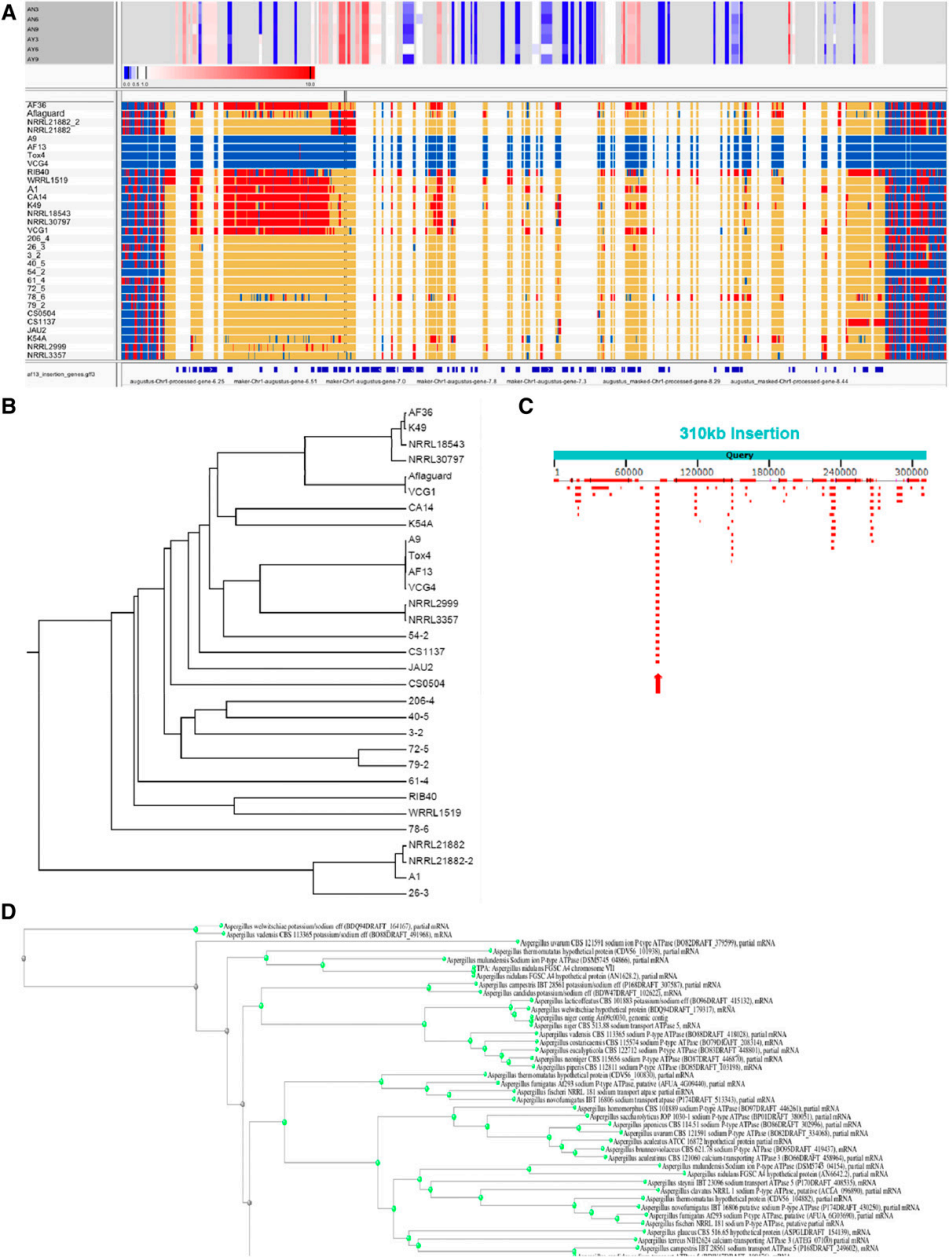
Variation in the 310Kb insertion gives insights into its origins and distribution within the species *Aspergillus flavus*. A. SNP calls within the insertion were evaluated. In the SNP plot, blue – AF13 calls; red – Non-AF13 calls; and yellow – no calls. The bounds of the insertion are visually apparent as an extended row of yellow (‘no call’) in strains lacking the insertion. Above the SNP calls, gene expression levels are displayed in the heatmap with box size corresponding to the position of each annotated gene in the insertion. Transcript expression levels for the annotated genes within the insertion in AF13 in response to oxidative stress over time (0 – 9 hr) are indicated above the SNP plots according to the inset scale. The positions of annotated genes within the insertion can be seen in the lowermost track below the SNP plots. Partial insertions can be observed in several biological control isolates. B. Neighbor-joining tree based on genome-wide SNP calls. AF13 and related isolates appear polyphyletic to the other *A. flavus* isolates. C. A single conserved gene, a Na ATPase, was identified in the insertion shown by BLAST hit alignments relative to the insertion (note the stacked hits for this gene indicated by the red arrow). D. Hits from related *Aspergillus* species were used to build a neighbor-joining tree. Maximum homology was only 84.35%, and the tree suggests that the insertion may be ancestral to the speciation of *A. flavus* and *A. oryzae*.

Single nucleotide polymorphism (SNP) calling was performed relative to the AF13 genome for each isolate with a focus on the insertion and the sequences immediately surrounding it (Figure 2A; Figure S3). The isolates A9, Tox4, and VCG4 (likely clonal to Tox4), were identical to AF13 for all loci and SNP calls throughout the insert region. Most of the isolates, including NRRL3357, showed little to no alignment of their contigs with the insert region yielding no detectable SNP calls. However, several isolates showed alignment and SNP detection, but with calls being predominantly non-AF13. These calls were concentrated in the first 100 Kb of the insert region and could be observed in the isolates A1, AF36, CA14, K49, NRRL18543 (AF36), NRRL30797 (K49), VCG1, and WRRL1519. This region contains 23 genes including NADH oxidase, glycoside hydrolase pyruvate decarboxylase, and extracellular endo-1,5-alpha-L-arabinase. The three available sequences for Afla-Guard (Aflaguard-2, NRRL21882, and NRRL21882_2) also showed partial alignment within the first 100 Kb, but to a lesser extent than those previously mentioned. RIB40 showed alignment for a majority of the insert and exhibited both AF13 and non-AF13 SNP calls. Regions surrounding the insert were also identical to AF13 in A9, Tox4, and VCG4, however generally exhibiting an even mixture of AF13 and non-AF13 SNP calls in the remaining isolates.

Using these genome-wide variant calls, a tree was constructed using an unweighted pair group method with arithmetic means to visualize the genetic relationship among the isolates (Figure 2B). Rooting was done based on the NRRL21882 lineage based on results from unrooted trees. As expected, the related isolates segregated into their own respective clades such as AF36, K49, NRRL18543, and NRRL30797. AF13 and its related isolates A9, Tox4, and VCG4 shared a sister lineage to NRRL3357 in the tree. However, *A. flavus* NRRL3357 and *A. parasiticus* NRRL2999 paired into their own clade, as did *A. flavus* WRRL1519 and *A. oryzae* RIB40.

In addition to variant calls, BLASTN analysis yielded a number of hits with high coverage. Alignment of the hits resulted in the identification of a Na P-type ATPase, maker-Chr1-augustus-gene-7.0, that was seemingly conserved across several *Aspergillus spp*. (Figure 2C). A portion of the insert containing this gene, 2,874 bp in length, was then searched in the nr database with blastn. The results showed a hit for a region on Chromosome 3 in *A. flavus* NRRL3357 (CP044620) with 100% coverage and 84.35% identity. The same could be found with the NRRL3357 assembly presented here. The same was observed for *A. oryzae* RIB40 SC023 with 100% coverage and 84.18% identity. A similar hit could also be found for the original Sanger sequenced assembly for NRRL3357 AFLA_110050. Interestingly, a second hit for this gene could be found in a similar location on Chromosome 3 in AF13, augustus-Chr3-processed-gene-16.18. This gene shared a 99.38% identity with the AFLA_110050 gene in the NRRL3357 Sanger assembly and similar levels in the current NRRL3357 assembly. Using the distance tree tool associated with NCBI blastn, a neighbor-joining tree was generated based on the top 100 alignments to the Na P-type ATPase from the insert (Figure 2D). This tree showed the genes found on Chromosome 3 in NRRL3357 to share a clade with *A. oryzae* RIB40, *A. sojae* SMF134, *A. bombycis*, and *A. nomius* NRRL13137 while the query AF13 sequence from the insertion was more ancestral sharing a common ancestor with this clade of Chromosome 3 hits from these species.

### Diverse, unique genes identified between assemblies

**Figure 3:**
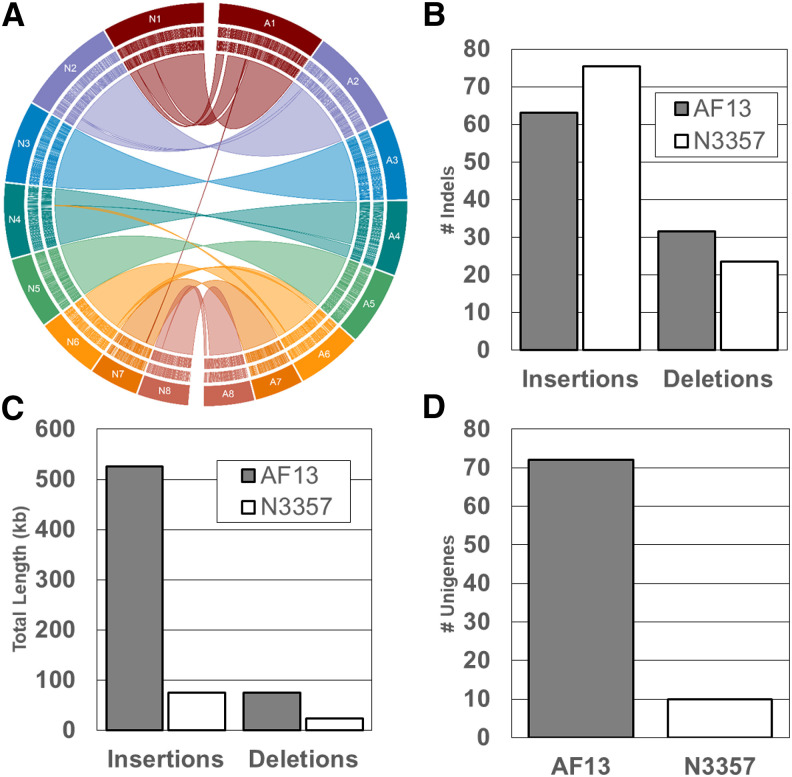
Indel analysis of the AF13 and NRRL3357 genome assemblies. A. Chromosome alignments between the assemblies showing indel locations. B. Insertion and deletion counts. C. Total length of the identified insertions and deletions in each assembly. D. Total number of indel-associated unique genes.

Given the distinct genetic relationship and observed phenotypes between AF13 and NRRL3357, the specific genes underlying these differences were investigated. Comparison of the two reference assemblies resulted in the identification of a number of unique genes (Figure 3; Figure S4). Based on indel analyses, AF13 was found to contain 153 unique genes interspersed throughout the genome. These genes could be subdivided into two groups, presence/absence and indel-associated genes. Among the 81 presence/absence genes (Table S3), most encoded for products involved in transmembrane transport, oxidation-reduction processes, and protein phosphorylation as indicated by GO biological process annotations. Of these genes, one Zn(II)_2_Cys_6_ transcription factor was identified along with the MAT1-2 mating type locus gene. In addition, benzoate 4-monooxygenase, S-adenosyl-L-methionine (SAM)-dependent methyltransferase, alkaline serine protease (PR1), and indoleamine 2,3-dioxygenase genes were also found among this group.

**Figure 4:**
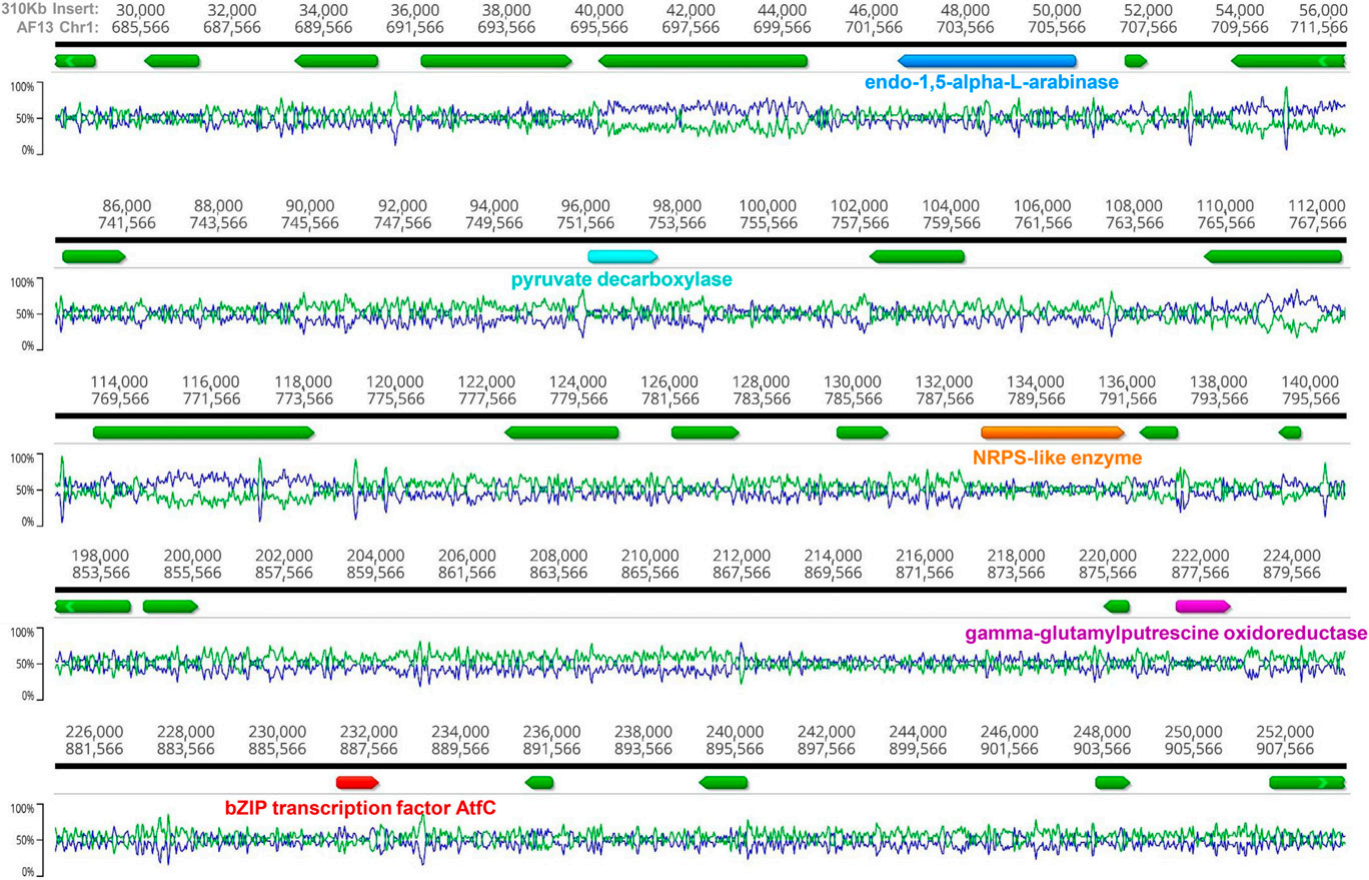
Composition and unique genes contained within the 310Kb insertion identified on Chromosome 1 of AF13. This plot of some select regions of the insertion contains colored arrows indicating genes of interest within the insertion. Relative position within the insertion and AF13 Chromosome1 are listed on the top of the plot. The line graphs show G/C (blue) and A/T (green) content along the sequence. A novel bZIP transcription factor, annotated here *atfC*, can be seen in red.

Of the 72 indel-associated genes unique to AF13 (Table S4), a majority were associated with a large 310 Kb insertion on Chromosome 1 (Figure 4). This insertion contains 60 genes of which 26 were expressed with an FPKM ≥ 2 in AF13 under oxidative stress over time in at least one replicate (Table S5). Differential expression analyses (Figure 2A, Table S5) identified 11 differentially expressed genes which were mostly up-regulated early in response to stress, but then leveled off over time. Among these genes, gamma-glutamylputresine oxidoreductase was found to be slightly down-regulated by oxidative stress while a cyclin-dependent kinase regulator Pho80, and a hypothetical protein AFLA70_740g000270 were significantly up-regulated by stress. The insert also included a novel non-ribosomal polyketide synthetase (NRPS)-like gene, however this gene was not expressed in the examined conditions. Also of interest were several constitutively expressed genes including a pyruvate decarboxylase, an extracellular endo-1,5-alpha-L-arabinase, and a novel bZIP transcription factor (augustus-Chr1-processed-gene-8.26-mRNA-1) putatively annotated here as *atfC*. The remaining indel-associated genes outside the insertion were dispersed among loci on Chromosomes 3 (1), 4 (9), 5 (2), 6 (1), and 8 (1). Genes of interest included a novel polyketide synthase, alanine racemase TOXG, and an acetyl-CoA synthetase-like protein gene.

In comparison to AF13, NRRL3357 was found to contain fewer (45) unique genes. Among the 35 presence/absence genes (Table S6), GO analysis showed enrichment for oxidation-reduction and transcriptional regulation among the genes. These genes included three that encoded transcription factors: a Zn(II)_2_Cys_6_, a C_6_ (Fcr1), and a C_2_H_2_ transcription factor. In addition to these, other genes of interest included a synaptic vesicle transporter and a dihydrofolate reductase. Among the 10 indel-associated genes (Table S7), genes of interest included those encoding for a C_6_ zinc finger protein, formiminoglutamate hydrolase, copper amine oxidase, and 1-aminocyclopropane-1-carboxylate oxidase (ACC). All of these genes are located on Chromosome 5 inside a 19Kb insertion.

### Secondary metabolite gene clusters

**Figure 5:**
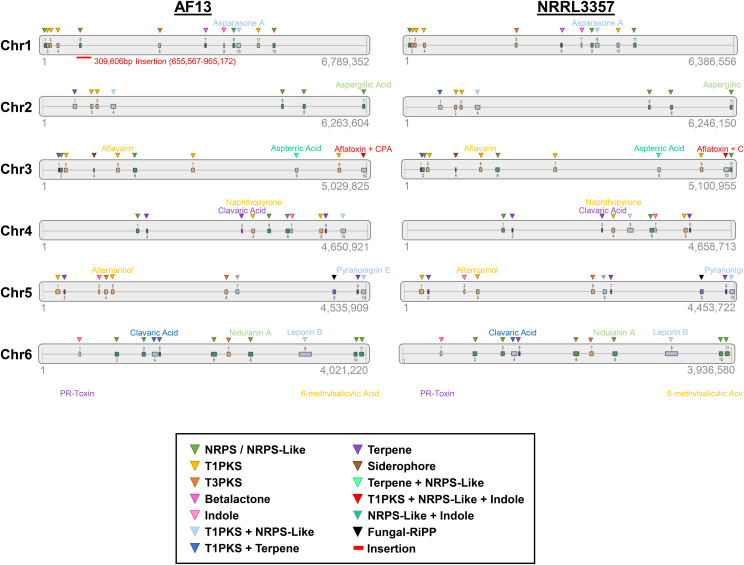
Secondary metabolite gene cluster prediction in the AF13 and NRRL3357 assemblies. Physical positions of secondary metabolite biosynthetic gene clusters identified by antiSMASH are plotted on each chromosome of the assemblies (gray bars, not to scale). The location and type of the core biosynthetic gene identified in each cluster are indicated by the colored triangles according the legend. The location of the 310 Kb insertion on AF13 Chromosome 1 is indicated by a red bar and associated text. Annotations of several secondary metabolites of interest identified by the analysis are listed above the triangles denoting their positions. Numbers below each chromosome plot indicate the lengths of each chromosome.

To identify secondary metabolite gene clusters present in the assemblies, antiSMASH was used to identify core biosynthetic genes within each assembly (Figure 5, Tables S8 and S9). The AF13 assembly contained 80 secondary metabolite gene regions consisting of 36 non-ribosomal polyketide synthetase (NRPS), 29 type 1 polyketide synthase, 13 terpene, 6 indole, 4 type 3 polyketide synthase, and one each of betalactone, fungal ribosomally-synthesized and posttranslationally-modified peptides (RiPP), and siderophore genes. Likewise, the NRRL3357 assembly contained 78 secondary metabolite gene regions consisting of 36 non-ribosomal polyketide synthetase (NRPS), 28 type 1 polyketide synthase, 15 terpene, 7 indole, 3 type 3 polyketide synthase, and one each of betalactone, fungal RiPP, and siderophore genes. Of the detected secondary metabolite core biosynthetic genes, five were unique to AF13 occurring on Chromosomes 1, 4, 5, and 7; and three were unique to NRRL3357 occurring on Chromosomes 1, 3, 4, 5, and 7. While most encoded for unknown gene products, the unique NRPS and type 1 polyketide synthase genes on Chromosome 4 of AF13 are homologous to citrinin biosynthetic genes. In NRRL3357, the unique terpene metabolite gene located on Chromosome 5 encodes for a geranyl-geranyl pyrophosphate synthase which is involved in the synthesis of several key precursor compounds for the production of terpenoid secondary metabolites, though the specific metabolite this gene is associated with is unknown.

#### atfC, a novel bZIP transcription factor gene in A. flavus:

**Figure 6:**
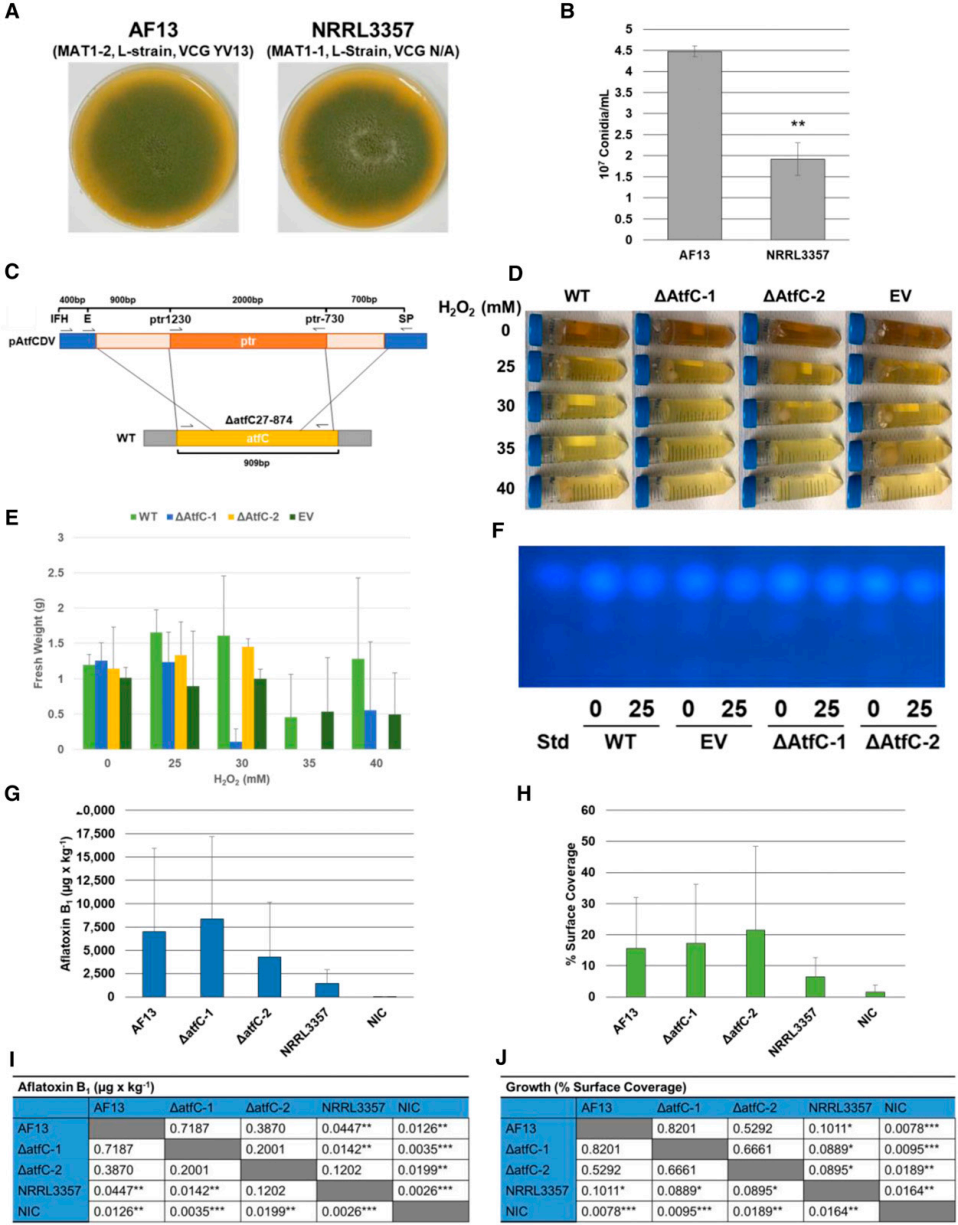
Isolate phenotypic evaluations and effects of the deletion of *atfC* in AF13 on oxidative stress tolerance and pathogenicity. A. Wild type AF13 (WT) and NRRL3357 cultures on V8 agar. B. Conidia counts AF13 and NRRL3357 conidial suspensions. NRRL3357 produced significantly fewer conidia than AF13. C. A double recombination strategy was employed for the deletion of the wild type *atfC* gene in AF13. This is elaborated on in Figure S5. D & E. Deletion mutants of *atfC* were grown in the dark with shaking at 150 rpm for five days in yeast-extract sucrose (YES) medium supplemented with increasing levels of H_2_O_2_ and compared with growth of AF13 (WT) and empty vector (EV) controls. Mycelia fresh weights indicated compromised oxidative stress tolerance in the mutant isolates, particularly for ΔatfC-2. F. Aflatoxin production was examined using thin layer chromatography (TLC) and no significant effects on aflatoxin were observed in the mutant isolates. G. Kernel screening assay (KSA) on the peanut cultivar Tifrunner. Comparison of the isolates (I) showed that AF13 had significantly greater aflatoxin production compared to NRRL3357. Mutant ΔatfC-2 showed aflatoxin levels comparable to NRRL3357 suggesting compromised aflatoxin production. H. Fungal growth in terms of percentage of kernel surface area covered by visible conidia. AF13 and the mutants showed marginally significantly more growth than NRRL3357 (J). In I and J, p-values are the results of two-tailed T-tests assuming equal variance. *P ≤ 0.10; **P ≤ 0.05; ***P ≤ 0.01.

The putative bZIP transcription factor gene a*tfC* identified within the 310Kb insertion in AF13 shares 74.84% similarity with the NRRL3357 *atf21* gene (*atfB*; AFLA_094010). Given the similarity between this novel transcription factor and *atf21*, which has been shown to coordinate secondary metabolism and aflatoxin production along with stress responses and developmental processes in *Aspergillus spp*. (Roze *et al.* 2011, 2013), functional analyses were performed to determine the potential function of *atfC*. Two independent deletion mutant isolates were generated, ΔatfC-1 and ΔatfC-2 (Figure S5). No gross morphological differences were observed when culturing the mutant isolates on V8 agar (Figure S6). However, obvious phenotypic differences including aerial mycelial growth and differences in conidia production can be observed between WT AF13 and NRRL3357 (Figure 6A,B). These mutants were evaluated for aflatoxin production and oxidative stress tolerance by culturing them on YES medium amended with different concentrations of H_2_O_2_ ranging from 25 to 45 mM for five days in the dark. This range was selected based on previously observed oxidative stress tolerance ranges for AF13 (Fountain *et al.* 2015a). There were no observable effects on aflatoxin production in ΔAtfC-1 and ΔAtfC-2 compared to wildtype AF13 (WT) and the empty vector (EV) control (Figure 6C). The H_2_O_2_ gradient study showed a significant reduction in fungal biomass under increasing levels of oxidative stress in ΔAtfC-1 and ΔAtfC-2 in comparison to the WT and EV controls when cultured in 50mL conical tubes with shaking (Figure 6D, E). However, this reduction was not observed consistently when culturing in 125 mL Erlenmeyer flasks which showed little differences between the mutant and control isolates representing possible artifacts in the system (Figure S7). In addition, observed growth of ΔAtfC-1 at 40mM was unexpected given it was completely inhibited at 35mM (Figure 6E). These observations may indicate possible escape of inoculum from H_2_O_2_ stress, and represent a possible artifact of the system.

In addition to stress responsiveness, the mutant isolates were also evaluated for plant pathogenicity and aflatoxin production during peanut kernel colonization. The mutant isolates ΔatfC-1 and ΔatfC-2, the wild type (WT) AF13, and NRRL3357 were inoculated onto seeds of the peanut cultivar Tifrunner which is moderately susceptible to *A. flavus* infection with increased aflatoxin contamination (Figure 6G, I). A non-inoculated control was also included as a reference for possible latent *A. flavus* seed infections. As a baseline comparison, AF13 and NRRL3357 were evaluated and AF13 was found to exhibit greater levels of kernel colonization, moderately significant with *P* = 0.1011, and aflatoxin contamination, significant with *P* = 0.0447, in comparison to NRRL3357. The ΔatfC-1 and ΔatfC-2 mutant isolates showed somewhat contrasting phenotypes with ΔatfC-1 exhibiting near WT levels of aflatoxin production while ΔatfC-2 showed reduced aflatoxin contamination similar to that observed for NRRL3357. Neither mutant event showed a significant effect on fungal colonization, but a marginally significant difference could be observed between AF13 and the mutants, and NRRL3357 (Figure 6H, J).

## Discussion

These assemblies represent a significant improvement in quality in comparison to the original scaffold-level reference genome for NRRL3357 (GCA_000006275.2) (Nierman *et al.* 2015; Payne *et al.* 2006; 2007; Yu *et al.* 2008). While some individual scaffolds of the previously assembled genome represented arms of chromosomes based on comparisons with *A. oryzae* (Machida *et al.* 2005; Payne *et al.* 2006), the current assembly has allowed for a complete picture of the full-length chromosome of NRRL3357. In comparison to the recently released NRRL3357 assembly by UC Berkeley (Skerker *et al.*, GCA_009017425.1), those presented here share comparable lengths for both individual chromosomes, 6.387 – 3.033 Mb in the present assembly and 6.510 – 3.252 Mb in the UC Berkeley assembly, and overall, 36.996 Mb in the present assembly and 37.749 Mb in the UC Berkeley assembly.

In addition to NRRL3357, AF13 was also used to generate a chromosome-arm genome assembly. This isolate is distinct from NRRL3357 in several areas. First, this isolate is from a distinct geographical and cropping system origin. AF13 was originally isolated from cotton field soils in Yuma Valley, Arizona, USA (Cotty 1989) while NRRL3357 was isolated from peanuts with visible mold in Georgia, USA (Wicklow and Shotwell 1983). Second, these isolates represent distinct mating types and vegetative compatibility groups (VCGs) with AF13 being MAT1-2 and a member of VCG YV13 while NRRL3357 is a MAT1-1 isolate with an as yet unreported VCG classification (Chang *et al.* 2012; Cotty 1989; Ehrlich *et al.* 2007; Olarte *et al.* 2013). Finally, these isolates display contrasting growth behaviors and aflatoxin production capabilities in *in vitro* assays. Here, AF13 was shown to exhibit significantly greater levels of aflatoxin production during *in vitro* seed colonization assays than NRRL3357 (Figure 6). The AF13 genome assembly was comparable to these other assemblies with a total size of 37.439 Mb. Lengths of individual chromosomes were also similar with the other assemblies ranging from 6.387 to 3.033 Mb (Table 1). However, the primary differences between these genomes came in terms of unique gene content. The AF13 assembly contained 153 unique genes compared to only 45 unique genes in NRRL3357 (Figure 3). These contrasting phenotypes and novel gene content make AF13 a useful and novel reference genome candidate and will prove useful for future studies.

Unique gene content in AF13 was also of particular interest given the observed higher levels of seed colonization and aflatoxin production in comparison to NRRL3357 (Figure 6). AF13 has been previously shown to exhibit high levels of maize pathogenicity (Guo *et al.* 1995; Fountain *et al.* 2015b; Mellon *et al.* 2005), and a high degree of oxidative stress tolerance (Fountain *et al.* 2015a). Of the presence-absence and indel-associated unique genes in AF13, a benzoate 4-monooxygenase gene (maker-Chr5-augustus-gene-38.22), an indoleamine 2,3-dioxygenase (augustus-Chr6-processed-gene-39.60), an acetyl-CoA synthetase-like protein (augustus_masked-Chr4-processed-gene-43.76), and an alanine racemase TOXG (augustus_masked-Chr4-processed-gene-43.29) gene were of interest for their potential roles in stress responses and mycotoxin production.

Benzoate-4-monooxygenase, a cytochrome p450 monooxygenase, was previously found to be up-regulated in AF13 in response to H_2_O_2_-induced oxidative stress (Fountain *et al.* 2016a, 2016b). Aminobenzoate derivatives including methyl benzoate, ethyl benzoate, salicylic acid, and *trans*-cinnamic acid have been demonstrated to inhibit both growth and aflatoxin production in *A. flavus* cultures (Chipley and Uraih 1980). Therefore, degradation of benzoic acid by this monooxygenase in AF13 in addition to mechanisms present in other loci in the genome may partially account for the increased level of aflatoxin production observed in AF13 compared to NRRL3357. Indoleamine 2,3-dioxygenase functions as an initial reaction in the catabolism of tryptophan to kynurenine. Previously we showed that NRRL3357 had significant increases in kynurenine accumulation in response to oxidative stress over time (Fountain *et al.* 2019b). Inhibition of kynurenine catabolism by kynurenine 3-monooxygenase has been shown to result in increased oxidative stress tolerance in fungi (Zhang *et al.* 2018). Presence of an additional copy of this gene may contribute to increased stress tolerance in AF13 under certain conditions. For the acetyl-CoA synthetase, acetyl CoA serves as the primary substrate used for the production of polyketide mycotoxins like aflatoxin (Abdollahi and Buchanan 1981; Buchanan and Ayres 1977). The presence of an additional copy of this gene in AF13 may also contribute to increased levels of aflatoxin production (Figure 6). Finally, the alanine racemase TOXG gene is a component of HC toxin production, a mycotoxin that has been shown to be involved in maize pathogenicity in *Cochliobolus carbonum* (Walton 2006). Comparison of the sequence of this gene by blastn showed significant homology only to *Uncinocarpus reesii* (Coverage: 93%, ID: 74.62%), *Coccidioides posadasii* (Coverage: 78%, ID: 69.71%), and *Coccidioides immitis* (Coverage: 78%, ID: 69.61%). No significant homologs could be found among the Aspergilli. This is interesting given that *C. posadasii* and *C. immitis* are both the causal agents of San Joaquin valley fever (coccidiodomycosis), and are endemic to the Southwestern US (Cole and Hung 2001). The model *U. reesii* is a non-pathogenic species used for studying *C. posadasii*, *C. immitis*, and related pathogens (Pan *et al.* 1994). This may provide for enhanced pathogenicity in AF13 for maize colonization. It also suggests that the TOXG-containing insertion on Chromosome 4 has been acquired by horizontal gene transfer (HGT) from a *Coccidioides sp*. given their co-localization both to soil environments, and to the Southwestern US in origin (Cotty 1989). Novel secondary metabolite clusters identified in AF13 may provide similar advantages, however, none of the detected novel clusters had a defined function based on homology to those in public databases (Figure 5).

The starkest finding of the indel and structural comparative analyses between the assemblies was the identification of a large 310 Kb insertion unique to Chromosome 1 of AF13. This insertion contained diverse assortment of genes including those encoding a gamma-glutamylputrescine oxidoreductase (*puuB*, augustus-Chr1-processed-gene-8.25), and a novel bZIP transcription factor (*atfC*, augustus-Chr1-processed-gene-8.26). The *puuB* gene functions in the degradation of putrescine, a polyamine compound that serves as a precursor for the biosynthesis of spermidine and spermine. Recycling of putrescine to succinate would allow for its use in energy metabolism, however this gene was found to be significantly downregulated in AF13 in response to oxidative stress (Figure 2A). Preventing putrescine degradation may promote additional spermidine and spermine production, both of which have been shown to accumulate in response to oxidative stress in *A. flavus*, and to be required for normal growth, development, and aflatoxin production in *in vitro* assays (Fountain *et al.* 2019b; Majumdar *et al.* 2018). Polyamine metabolism here may also be connected to the previously described benzoate-4-monooxygenase system. The product of benzoate-4-monooxygenase, 4(p)-hydroxybenzoate, is also a precursor of folate biosynthesis which feeds the biosynthesis of SAM, a regulator of polyamine metabolism (Bistulfi *et al.* 2009; Lozoya *et al.* 2018). Therefore, polyamine metabolism may form the basis of a significant antioxidant mechanism employed to a greater extent in AF13 and warrants further investigation.

The novel bZIP transcription factor, annotated here as AtfC, shares homology with the previously characterized *A. flavus* bZIP transcription factors AtfA (51.19%) and Atf21/AtfB (74.84%). These transcription factors have been shown to regulate the production of aflatoxin and its precursors in response to oxidative stress in *Aspergillus spp*., and to coordinate oxidative stress responsive genes including catalase (Baidya *et al.* 2014; Roze *et al.* 2011, 2013). Silencing of *atfA* expression in *A. nidulans* has been shown to compromise tolerance to oxidative stress induced by several compounds including H_2_O_2_, menadione sodium bisulphite, and tert-butylhydroperoxide (Balázs *et al.* 2010; Emri *et al.* 2015). Silencing of *atfB* expression in *A. parasiticus* was also shown to compromise aflatoxin cluster and virulence-related gene expression and inhibit conidia production (Wee *et al.* 2017). Previously, the expression of these genes was observed to increase in NRRL3357 and AF13 in response to increasing oxidative stress at later timepoints in culture (Fountain *et al.* 2016b). Taking these facts together, therefore, the possibility of expression of a third as yet undescribed activating transcription factor (ATF) warrants investigation in AF13. Silencing of *atfC* in AF13 resulted in compromised oxidative stress tolerance to a varying degree between assays and the generated mutants (Figure 6) and had no obvious morphological effect in comparison to the WT or the empty vector control isolates (Figure S6). The ΔatfC-2 mutant did show a significant reduction in aflatoxin production in the kernel assay in comparison to the WT AF13 isolate to a level comparable to NRRL3357 (Figure 6). This suggests that AtfC may act as a supplement to the transcriptional regulation provided by AtfA and Atf21/AtfB, though the specific mechanism as to how this is accomplished is unknown and requires further investigation.

The prevalence of this potentially advantageous insertion was investigated among the available genome assemblies for *A. flavus* and closely related species including *A. oryzae* and *A. parasiticus*. The genomes of 10 additional isolates (nine *A. flavus* and one *A. parasiticus*) were sequenced and used for the evaluation of diversity within the insert and genome-wide (Fountain *et al.* 2020) along with several obtained from NCBI (Table S1). Plotting SNPs along the insertion, clear patterns can be observed regarding AF13 and non-AF13 calls which point to the distribution of some portions of the insertion among the examined isolate genomes (Figure 2A). However, careful examination showed that only the first ∼100 Kb of the insertion were present mainly in atoxigenic biological control isolates such as AF36 (NRRL18543), K49 (NRRL30797), and WRRL1519 (Chang *et al.* 2012; Pennerman *et al.* 2018; Yin *et al.* 2018), and even then exhibited significant levels of polymorphism compared to AF13 and its related isolates. This region contained several genes involved both in energy production, defense responses, and in the catabolism of pectin, all of which are potentially beneficial to saprophytic and plant pathogenic fungi. Therefore, this region may contribute to the efficacy of these isolates as biological controls in competition with native aflatoxigenic *A. flavus* populations in field environments.

In examining the insertion for orthologs in other *Aspergillus spp*. by blastn analysis, it was found that a single Na ATPase gene (maker-Chr1-augustus-gene-7.0) was conserved across multiple Aspergilli and was used for construction of neighbor-joining tree (Figure 2B). This gene, which has a homolog on Chromosome 3 in both AF13 and NRRL3357, is distinct from its orthologs within the genus. The relatively low degree of homology with *A. flavus* and *A. oryzae*, which shared the most homology overall for the insertion on Chromosome 8 of the *A. oryzae* RIB40 genome (Figure 1), does suggest that this gene, and therefore the insertion, may be ancestral to speciation between *A. flavus* and *A. oryzae*, and preserved at least in part in lineages of both species. This assertion is further supported by the examination of genome-wide variants and the construction of a rooted neighbor-joining phylogenetic tree in this analysis (Figure 2B). Here, *A. oryzae* RIB40 and most *A. flavus* clades diverged after the separation of the NRRL21882 lineage. Given that NRRL21882 contains only a small portion of the insertion, it seems likely that in the other clade containing AF13 and RIB40, the insertion was preserved being passed along in part to the AF36 clade and to the AF13 clade, and not to the remainder including NRRL3357. This may also be true for the aflatoxin gene cluster, and not only for the insertion given that all the members of the NRRL21882 clade are atoxigenic isolates while the remaining isolates and species within the tree contain at least partial aflatoxin clusters (Chang *et al.* 2005; Faustinelli *et al.* 2016; 2017).

Surprising here is the level of similarity observed between *A. flavus* NRRL3357 and *A. parasiticus* NRRL2999, and between *A. flavus* WRRL1519 and *A. oryzae* RIB40 (Figure 2B). This close relationship is supported in the literature with WRRL1519 having been previously reported to be more genetically related to *A. oryzae* than other *A. flavus* isolates (Chang 2019). This same report by Chang (2019) also supports the hypothesis that NRRL21882 is more genetically related to *A. oryzae* compared to other toxigenic L-strains of *A. flavus* which concurs with the phylogenetic analysis here. At the genus level, *Aspergillus* has been clearly demonstrated to be monophyletic in relation to other related members of the Eurotiales and Trichocomaceae such as *Penicillium* (Frisvad *et al.* 2019; Kocsubé *et al.* 2016; Samson *et al.* 2014). However, within the species there has been more variation in classification over time with *A. oryzae* and *A. parasiticus* being previously referred to as subspecies within *A. flavus* (Kurtzman *et al.* 1986). Distinctions based on morphological characteristics in addition to sequencing of conserved genes such as internal transcribed spacer (ITS) rRNA sequences have since been used to classify these as distinct species from *A. flavus* (Kumeda and Asao 1996; Machida *et al.* 2008; Peterson 2008; Varga *et al.* 2011). Making this distinction, the tree presented here concurs and supports the proposal that *A. flavus* is comprised of a polyphyletic collection of related isolates, subspecies, and species as presented in the literature (Chang *et al.* 2006; Chang 2019; Geiser *et al.* 1998, 2000; Gonçalves *et al.* 2012; Moore *et al.* 2009; Okoth *et al.* 2018). However, the close relationship of these distinct species with *A. flavus* isolates described in the present study from the genomics perspective does cast doubt on the classification of these as distinct species rather than as subspecies of *A. flavus*. In comparison to ITS, whole genome sequencing allows for the evaluation of evolutionary changes throughout the entire genome, and should result in increased statistical power to delineate species and subdivisions within them (Baumsteiger *et al.* 2017). Addressing these classification issues will require the increasing prevalence of genomics information for isolates within this species, and studies comparing the results of genomics analyses and traditional ITS barcoding along with evaluating the reliability of common morphological characteristics for use in delineating species.

In conclusion, these newly generated, high quality, reference genomes for AF13 and NRRL3357 will provide new tools in the toolbox for genomics-assisted research into these important fungi. Comparative genomics analyses here have also identified genes and components of these isolate genomes which may contribute to plant pathogenicity, aflatoxin production, and biocontrol efficacy. They also provide a foundation for the beginnings of a pangenomic understanding of *A. flavus* by providing insights into novel gene content and structural variants which do not present in the previous reference isolate, NRRL3357. This novel gene content may prove useful in the elucidation and development of host resistance mechanisms against *A. flavus* colonization, biological control selection and screening, and field and storage-focused control measures to mitigate aflatoxin contamination.
